# Integrative EEG biomarkers predict progression to Alzheimer's disease at the MCI stage

**DOI:** 10.3389/fnagi.2013.00058

**Published:** 2013-10-03

**Authors:** Simon-Shlomo Poil, Willem de Haan, Wiesje M. van der Flier, Huibert D. Mansvelder, Philip Scheltens, Klaus Linkenkaer-Hansen

**Affiliations:** ^1^Department of Integrative Neurophysiology, Center for Neurogenomics and Cognitive Research, VU University AmsterdamAmsterdam, Netherlands; ^2^Department of Clinical Neurophysiology and MEG, VU University Medical CenterAmsterdam, Netherlands; ^3^Department of Neurology, Alzheimer Center, VU University Medical CenterAmsterdam, Netherlands; ^4^Department of Epidemiology and Biostatistics, VU University Medical CenterAmsterdam, Netherlands

**Keywords:** Neurophysiological Biomarkers, Alzheimer's disease, mild cognitive impairment (MCI), electroencephalography, predictive analysis, time series analysis, eyes closed resting state

## Abstract

Alzheimer's disease (AD) is a devastating disorder of increasing prevalence in modern society. Mild cognitive impairment (MCI) is considered a transitional stage between normal aging and AD; however, not all subjects with MCI progress to AD. Prediction of conversion to AD at an early stage would enable an earlier, and potentially more effective, treatment of AD. Electroencephalography (EEG) biomarkers would provide a non-invasive and relatively cheap screening tool to predict conversion to AD; however, traditional EEG biomarkers have not been considered accurate enough to be useful in clinical practice. Here, we aim to combine the information from multiple EEG biomarkers into a diagnostic classification index in order to improve the accuracy of predicting conversion from MCI to AD within a 2-year period. We followed 86 patients initially diagnosed with MCI for 2 years during which 25 patients converted to AD. We show that multiple EEG biomarkers mainly related to activity in the beta-frequency range (13–30 Hz) can predict conversion from MCI to AD. Importantly, by integrating six EEG biomarkers into a diagnostic index using logistic regression the prediction improved compared with the classification using the individual biomarkers, with a sensitivity of 88% and specificity of 82%, compared with a sensitivity of 64% and specificity of 62% of the best individual biomarker in this index. In order to identify this diagnostic index we developed a data mining approach implemented in the Neurophysiological Biomarker Toolbox (http://www.nbtwiki.net/). We suggest that this approach can be used to identify optimal combinations of biomarkers (integrative biomarkers) also in other modalities. Potentially, these integrative biomarkers could be more sensitive to disease progression and response to therapeutic intervention.

## Introduction

Caused by an increasing average age of the population in the developed world, dementia is becoming a major healthcare problem. Alzheimer's disease is the most common form of dementia and the golden standard for diagnosis is the post-mortem identification of Amyloid Beta 42 depositions and tangles (Blennow et al., 2006; Herrup, 2010). It has been suggested that Alzheimer's disease begins years, maybe even decades before actual cognitive symptoms appear (Sperling et al., 2011). However, normal ageing is also characterized by a slow decline of cognitive functions, which means it can be difficult to disentangle normal ageing from Alzheimer at a very early stage.

Patients with mild cognitive impairment (MCI) are at high risk of developing Alzheimer's disease. The label MCI is given when there is a cognitive complaint (mostly memory), which can also be demonstrated on formal testing, while general cognitive functioning is relatively intact and a patient is still living independently (Flicker et al., 1991; Gauthier et al., 2006; Albert et al., 2011). Therapies that stop the conversion to Alzheimer's disease unfortunately remain to be developed, but it is likely that these drugs or therapies will appear in the future (Prins et al., 2010; Huang and Mucke, 2012). It is plausible that these therapies will be most effective before major brain damage has occurred and it is, therefore, important to develop biomarkers sensitive of this very early stage (Sperling et al., 2011). Early-stage identification may also help the development of new treatments that are more effective at this stage as it can facilitate monitoring of the response to the intervention.

**Figure 1:**
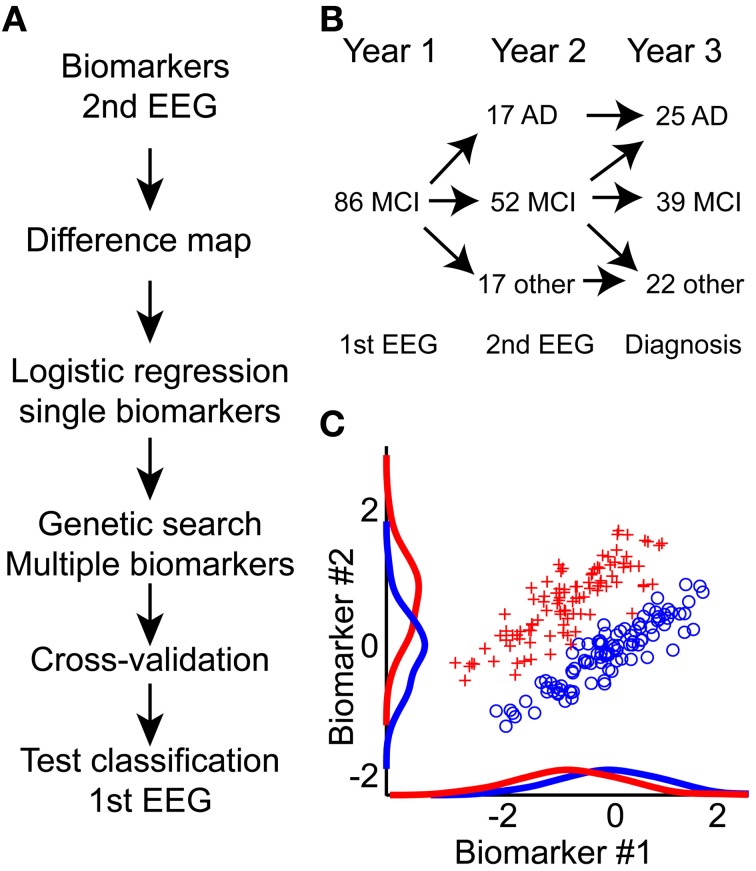
**An integrative approach toward improved prediction of mild cognitive impairment to Alzheimer's disease conversion. (A)** Diagram of processing flow. We calculate biomarkers on the second year EEG recording; hereafter we mapped all potential differences between MCI and AD using Student's *t*-test (Difference map). Next, we performed logistic regression on each single biomarker. Biomarkers with best single-classification power were seeded to a genetic search algorithm; this algorithm further optimized the combined biomarker set. This biomarker set was then used to predict MCI to AD conversion based on the first-year EEG recording. To evaluate the lower bound on the classification, half-split cross-validation was performed. Finally, the outcome performance was evaluated on the 1st EEG recording. **(B)** Overview of how the MCI patient cohort splits into AD, another diagnosis, or remain MCI one or 2 years after the in-take. **(C)** The integration of multiple biomarkers can reveal hidden separation boundaries. Here, we show two simulated biomarkers where the red and blue groups are overlapping if we only consider the single biomarkers. By combining the biomarkers, we see a clear separation boundary at the diagonal. Classification algorithms aim to identify this boundary, and use it to predict group association for new data.

We here focus on biomarkers obtained from electroencephalography (EEG) recordings in the eyes-closed resting state (ECR). EEG biomarkers are optimal for screening purposes because the EEG recording can be obtained using relative cheap and non-invasive equipment, which is widely available and fast to use. Several previous EEG studies of conversion from mild cognitive impairment to Alzheimer's disease have been conducted (Jelic et al., 1996, 2000; Huang et al., 2000; Stam et al., 2003; Schoonenboom et al., 2004; Rombouts et al., 2005; Babiloni et al., 2006, 2011; Kwak, 2006; Rossini et al., 2006, 2008; Lehmann et al., 2007; Moretti et al., 2007a, b, 2008, 2011; Luckhaus et al., 2008) mainly using biomarkers such as spectral measures and synchronization between brain regions. Machine-learning techniques have been used to explore differences between MCI and AD with varying success (Huang et al., 2000; Bennys et al., 2001; Prichep et al., 2006; Buscema et al., 2007; Lehmann et al., 2007; Prichep, 2007; Rossini et al., 2008), however, only few studies have tried to predict the conversion from MCI to AD (Prichep et al., 2006; Prichep, 2007; Antila et al., 2013). Many studies typically focus on a small number of biomarkers (on the order of 15 marker values), and some do not have adequate validation of their results on independent groups. We perform large-scale data mining of multiple biomarkers (Figure 1A) and validate our results on an independent group of subjects.

Our focus is on the EEG measured as part of the initial hospital intake test, combined with longitudinal recordings measured 1 year after the initial intake test. We have mapped several classical EEG biomarkers, such as frequency and power, but also non-classical biomarkers such as detrended fluctuation analysis and oscillation burst analysis (Poil et al., 2008; Montez et al., 2009). By combining several biomarkers, it is often possible to find better separation boundaries between two groups (Figure 1C), because each biomarker gives additional information (Lehmann et al., 2007). In this longitudinal study we show that EEG biomarkers from the initial hospital in-take test retrospectively can be used in a classifier algorithm to predict the diagnosis that the patient obtained within the subsequent 2 years.

## Methods and materials

### Subjects

The study involved 86 mild cognitive impairment (MCI) subjects who were referred to the Alzheimer Center at the VU University Medical Center in Amsterdam, the Netherlands (Figure 1B). Upon the first visit at the Alzheimer Center, all subjects underwent a thorough 1-day examination consisting of history taking, physical, and neurological assessment, neuropsychological testing including the Mini Mental State Examination (MMSE) (Folstein et al., 1975), laboratory tests, structural magnetic resonance imaging (MRI), and a routine electroencephalogram (EEG). After reviewing the clinical and ancillary imaging data, a multidisciplinary team established a consensus-based final diagnosis for each patient. The initial diagnosis of MCI was based on the criteria set by (Petersen et al., 1999), consisting of (a) objective memory impairment as seen during neuropsychological evaluation, defined by performances ≥ 1.5 standard deviation below the mean value of education—and that of age matched controls, (b) normal activities of daily living, and (c) a rating score of 0.5 in clinical dementia (Hughes et al., 1982).

All MCI subjects were followed up clinically during an average period of 709 ± [537:779] days (1.9 years) (median ± 95% confidence interval). The clinical follow up included medical history and functional status assessment re-examination in order to measure potential changes in the cognitive domain. MCI subjects who showed steady or enhanced cognitive functioning (but still fulfilled the criteria for MCI) during re-assessment were considered as MCI-stable, while MCI subjects who showed impoverished cognitive functioning, and fulfilled the NINDS-ADRDA criteria (McKhann et al., 1984) to be diagnosed with Alzheimer's disease, were considered to belong to the AD-converter group. Exclusion criteria were previous head trauma, history of neurological or psychiatric disease or use of psychotropic medications. Patients progressing from MCI to other disorders than Alzheimer's disease (*n* = 22) were excluded from the analyses reported here. These patients progressed to; “Subjective complaints” (*n* = 9), possible Alzheimer's disease (*n* = 1), frontal lobe dementia (*n* = 1), vascular dementia (*n* = 3), Lewy body dementia (*n* = 1), dementia other (*n* = 2), psychiatric (*n* = 2), or another neurological disorder (*n* = 3). The measurements were approved by the Ethics Committee of the VU University Medical Center, and were in accordance to the Helsinki declaration. All subjects signed an informed consent.

### EEG recordings

Twenty-one channel EEGs were recorded in a sound attenuated, electrically shielded, and dimly lit room. These recordings were performed with OSG digital equipment (Brainlab®) at the following locations of the international 10–20 system: Fp2, Fp1, FT9, FT10, F8, F7, F4, F3, A2, A1, T4, T3, C4, C3, T6, T5, P4, P3, O2, O1, Fz, Cz, and Pz. The recording was referenced to the common average of all electrodes, excluding Fp1 and Fp2. Sampling frequency was 500 Hz and analogue-digital precision was 16 bit. The impedance of all electrodes was less than 5 kΩ. Recordings were made with a 70 Hz low-pass filter (time constant 1 s). Subjects sat in a reclined chair for approximately 20 min. During this period the subjects kept their eyes closed most of the time, however, at irregular intervals, they were asked to open their eyes when drowsiness was noticed. Approximately 15 min into the recording a memory task, which consisted of remembering pictogram images for 1 min was performed.

### EEG cleaning

The recordings during task and eyes-open were not analyzed. The EEG was viewed in windows of 5 s, and sharp transient artifacts were cut out. On average 17.8 [range (12.4:24.1)] minutes of eyes-closed rest EEG was left. The JADE ICA algorithm was then used to separate the signal into 23 components (Cardoso and Souloumiac, 1993). Eye movements, eye blinks, muscle artifacts, and heartbeat components were rejected, based on abnormal topography, component activation, activity distribution, and spectrum.

### Biomarkers and processing flow

**Figure 2:**
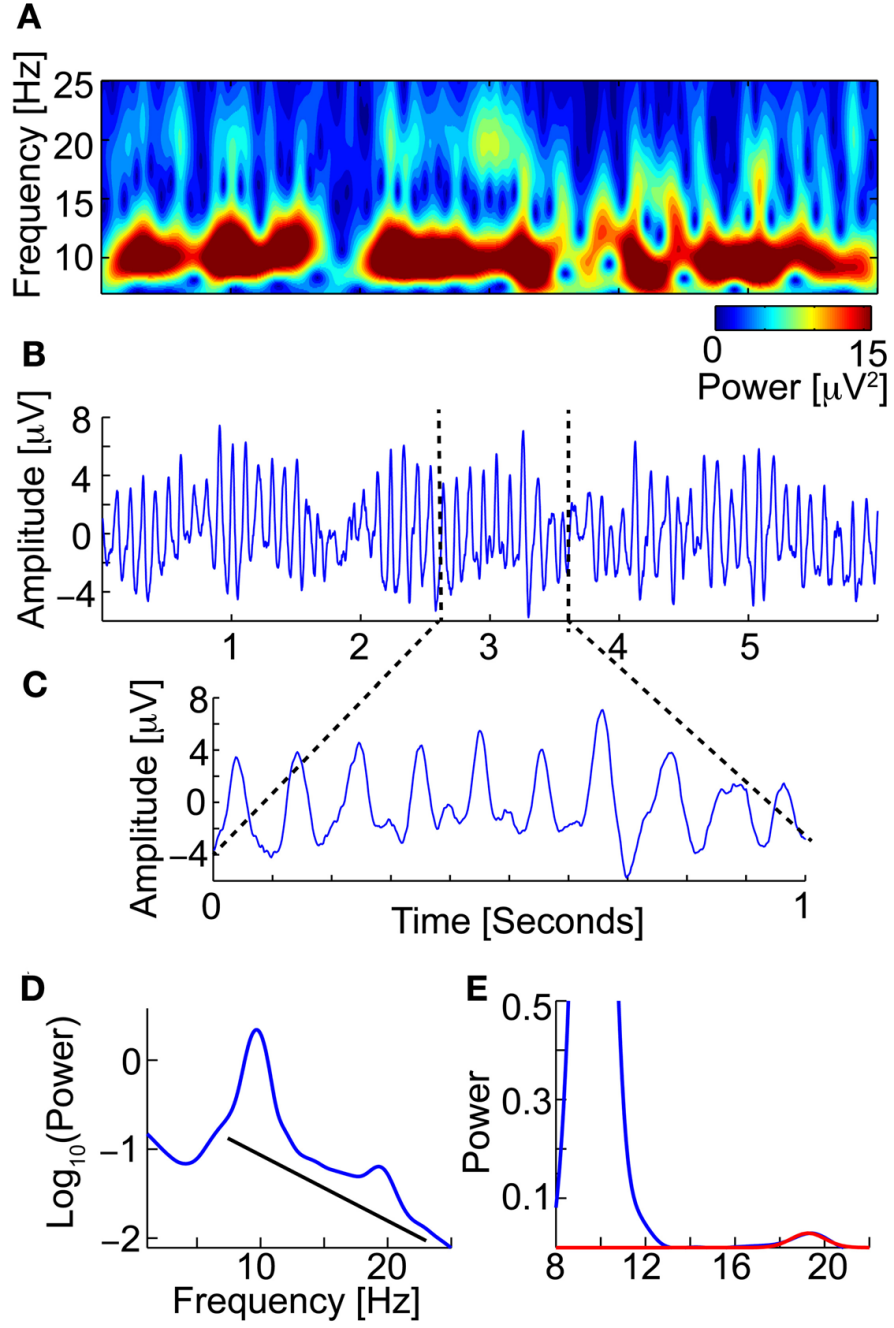
**An EEG biomarker is a quantitative measure derived from the EEG**. For example the Beta peak frequency. **(A)** Time-frequency (TF-plot) plot of 6 s of an eyes-closed rest EEG signal (from Pz) (Wavelet). The color shows the power. Low-amplitude bursts in the beta-frequency band (13–30 Hz) not directly coupled with the strong alpha are observed. **(B)** The raw EEG signal used to calculate the TF-plot in **(A)**. Clear and strong alpha (8–13 Hz) oscillations are observed. **(C)** Zooming in, we observe small peaks in the space between the strong alpha oscillation peaks, which correspond to the beta oscillations. **(D)** The power spectrum of the full-length EEG signal reveals a beta peak (left). To find the beta peak we first fit a 1/f baseline (right), next we fit a Gaussian to the small beta peak. We now have four biomarkers; Beta peak frequency, Beta peak width, Beta peak corrected power (i.e., minus 1/f baseline), and peak uncorrected power.

The Neurophysiological Biomarker Toolbox (NBT) (http://www.nbtwiki.net/) was used to organize, analyse, and calculate all biomarkers in this study (Hardstone et al., 2012). An EEG biomarker is a quantitative measure derived from the EEG, e.g., the dominant frequency of the beta frequency band (13–30 Hz), to be used as a diagnostic or prognostic predictor of disease (Figure 2).

We extracted 177 biomarkers from each EEG trace. We decided to focus on biomarkers we have had good experiences with in other studies, and acknowledge that many more biomarkers could have been selected.

Based on the broadband signal, we computed 28 biomarkers, namely: Hjorth's activity, mobility and complexity parameters (Hjorth, 1970); Time domain Parameters (Goncharova and Barlow, 1990), Wackermann's global field strength, global frequency, and spatial complexity (Wackermann, 1999), Barlow's amplitude, frequency and spectral purity (Goncharova and Barlow, 1990). Alpha peak frequency, peak width, power corrected for 1/f baseline (Poil et al., 2011), when applicable the same parameters where found for double alpha peaks. Alpha-theta transition point (Klimesch, 1999), Beta peak frequency (Figure 2), width, power corrected for 1/f baseline (Van Aerde et al., 2009), same for second beta peak if present; Frequency stability was evaluated using different methods, by the standard deviation and interquartile range of the central frequency and maximum wavelet frequency calculated in windows, and by, the distribution parameters of the phase values above zero, and of the number of oscillation cycle peaks per window.

For each of the classical frequency bands—delta (1–3 Hz), theta (4–7 Hz), alpha (8–13 Hz), beta (13–30 Hz), and gamma (30–45 Hz)—we computed 13 biomarkers; namely: The amplitude envelope was extracted using Hilbert transform and characterized extensively. We calculated the spearman correlations of amplitude envelopes in different channels. The distribution of amplitude values was characterized by kurtosis, skewness, interquartile range, median, range, and variance. Furthermore, detrended fluctuation analysis characterizing long-range temporal correlations (Linkenkaer-Hansen et al., 2001; Hardstone et al., 2012; Poil et al., 2012), multifractality spectral width (Kantelhardt et al., 2002; Ihlen, 2012) and oscillation bursts 95th percentile durations and sizes (Montez et al., 2009; Poil et al., 2011) were calculated on the amplitude envelope. The instantaneous phase was also extracted using Hilbert transform, and the 95th percentile duration and size of the stable phase bursts (a phase bursts is defined as the period between phase slips) were calculated. In addition, we computed for all frequency bands and individualized frequency bands, defined as Alpha1 (APF = individually defined Alpha peak frequency): (APF–4 to APF–2) Hz, Alpha2: (APF–2 to APF) Hz, Alpha3: (APF to APF+2) Hz; Beta: (APF+2 to 30) Hz (Klimesch, 1999), 7 biomarkers: absolute, relative power, and power ratios, furthermore, the central frequency, power in central frequency, bandwidth and spectral edge (Vural and Yildiz, 2010; O'Gorman et al., 2013). In total, we extracted 177 biomarker values from each EEG trace (Table 1).

**Table 1:** **Thirty-five biomarkers from different signal processing domains were extracted**.

**Spatial biomarkers**	**Temporal biomarkers**	**Spectral biomarkers**
Spearman correlations of the amplitude envelope across channels	Detrended fluctuation analysis	Absolute and relative power
	Multifractal spectral width	Central frequency
	Oscillation bursts duration and size	Power in central frequency
	Stable phase bursts duration and size	Bandwidth and spectral edge
	Frequency stability; standard deviation, interquartile range of central frequency, maximum wavelet frequency; distribution parameters of the phase values above zero; number of oscillation cycles per window	Hjorth's activity, complexity, and mobility
		Wackerman's Global Field strength, global frequency, and spatial complexity
		Barlow's amplitude, frequency, and spectral purity
	Amplitude envelope parameters; kurtosis, skewness, interquartile range, median, range, and variance	Alpha peak frequency, peak width
		Alpha peak power corrected for 1/f baseline
		Beta peak frequency, peak width
		Beta peak power corrected for 1/f baseline

Next, we performed data mining on these biomarkers based on the second EEG recording (Figure 1A), to identify biomarkers that reached a significance level of *p* < 0.05 (student's *t*-test) for the comparison of stable MCI vs. AD-converters (based on the diagnosis after 2 years). We here use student's *t*-test because this test has best statistical power in most cases under the assumption of normal distributed biomarker values. The biomarkers were tested per channel, and a binomial multiple-comparison correction was performed (Poil et al., 2011). The binomial multiple-comparison correction tests whether a significant number of channels are found (i.e., 3 or more channels, *p* < 0.05). The performance of two different classification algorithms (see below for details) in integrating significant biomarkers into a diagnostic index was then tested using their median values across significant channels.

### Development of a diagnostics index

To move beyond single-biomarker classification we aimed to integrate several EEG biomarkers in a diagnostic index that would classify the AD-converter group from the MCI-stable group better than each individual biomarker. Using one dataset for development and testing is not recommended, because it is theoretically possible to find a perfect separation of two groups if enough biomarkers are included (so-called over-fitting). To counteract this issue we build our classification model based on the second EEG recording (which was obtained in 34 out of a total of 64 subjects that were either MCI-stable or AD-converters), and tested the classification accuracy retrospectively on the first EEG recording. Thirty subjects were not included in the training (22 MCI-stable, 8 AD-converters), because these subjects did not have any second-year recording. These subjects serve as our ultimate classification test. We also used half-split cross-validation to evaluate the stability and lower bound of the solution (see below).

### Statistics: logistic regression with genetic search

Binary classification was performed using logistic regression. In logistics regression the binary outcome either AD-converter (1) or MCI-stable (0) is regressed with a linear combination of biomarkers. More specifically we fit a function *f*(*z*) using maximum likelihood.

$$f\left(z\right)=\frac{1}{\text{1}+e^{-z}}$$

with

(1) $$z=\beta_{0}+\sum_{i=\text{1}}^{k}\beta_{i}x_{i},$$

and *x* are the *k* biomarkers included in the regression (included as medians across significant channels), and β_*i*_ are the regression coefficients. The function *f* represents the probability of Alzheimer's disease. We use the 50% probability as our classification threshold, i.e., if *f* ≥ 0.5, the patient belong to the AD-converter group, otherwise the patient belongs to the MCI-stable group. We used a genetic search method to identify biomarkers that combined (using logistic regression) would give the best classification of the outcome MCI-stable vs. AD-converters. Genetic search is considered an efficient method for searching large data sets, instead of the computationally demanding alternative of testing all possible combinations (Koza and Poli, 2005; Zviling et al., 2005). The genetic approach is based around an evolutionary idea where the combined set of biomarkers is “mutated” by different mutation rules; addition of a random biomarker, removal of a biomarker, random selection of a new set of four biomarkers, and random substitution of a biomarker. Each rule was applied 5 times in each generation, leading to 20 new sets of biomarkers. The classifications of these new sets were then compared with the previous optimal set. Only the best biomarker set survived and was used as the base for next generation of mutations. We did not set limits on the maximum or minimum number of biomarkers in each set.

The genetic algorithm was seeded with an initial set of five biomarkers with the highest Matthew correlation coefficient (see outcome evaluation below). The genetic algorithm ran for 100 generations. At each generation the biomarker set with maximal positive likelihood ratio (see outcome evaluation below) survived. In all cases the logistic regression model was fitted using the second EEG recording, and the classification outcome was measured using the first EEG recording.

### Statistics: elastic net logistic regression

As an alternative to genetic optimization of biomarkers included in the logistic regression, we employed an elastic net logistic regression algorithm (Zou and Hastie, 2005) as implemented in the GLMnet package for Matlab (http://www-stat.stanford.edu/|tibs/glmnet-matlab/) (Friedman et al., 2010). This algorithm promises a build-in selection of features that optimally can perform much better than the less stable genetic optimization. The elastic net optimizes the number of biomarkers included in the diagnostic index by minimizing both the L1 and L2 norm of the regression coefficients by minimizing the equation

$$L\left(\lambda_{1},\lambda_{2},\beta \right)={\left|z-X\beta \right|}^{2}+\lambda_{1}\left|\beta \right|+\lambda_{2}{\left|\beta \right|}^{2}$$

where the first term is similar to the logistic regression, and the second and third are the penalizing terms (the elastic net) (Zou and Hastie, 2005). The parameters λ_1_ and λ_2_ determines the influence of either the L1 or L2 norm penalty. We define a new combined parameter

$$\alpha =\frac{\lambda_{2}}{\lambda_{1}+\lambda_{2}}$$

which we optimized in 5-split cross-validation based on the best classification by training on second-year data, and testing on the 1/5 left-out subject group on first-year EEG (note that subjects which did not have a second-year EEG were not included, and, therefore, serve as our ultimate test group (see Results) (data not shown). We found the best classification with α = 0.8.

### Statistics: classification outcome evaluation

To evaluate the outcome of our classification we use five different measures:

- Sensitivity (SE): defined as the (number of correctly classified AD-converter patients)/(number of AD-converter patients).
- Specificity (SP): defined as the (number of correctly classified MCI-stable subjects)/(number of MCI-stable subjects).
- Positive predictive value (PPV): defined as (number of correctly classified AD-converter patients)/(number of patients classified as AD-converters).
- Positive likelihood ratio (PLR): defined as (Sensitivity)/(1-Specificity).
- Matthew correlation coefficient (MCC): explains the correlation between the outcome and the expected outcome (Baldi et al., 2000).

A Matthew correlation coefficient higher than 0.20, sensitivity higher than 65%, specificity higher than 65%, positive predictive value higher than 65%, and a positive likelihood ratio higher than 1.6 means that the classification is significantly different from a random classification (Monte Carlo simulation, 5000 iterations, *n* = 65, note these results depends on the sample size making the threshold levels lower for larger sample sizes, *p* < 0.05). Perfect classification would give a Matthew correlation coefficient (MCC) of 1, sensitivity of 100%, specificity of 100%, positive predictive value of 100%, and an infinite positive likelihood ratio.

An issue with these outcome measures is that they only tell how well the classification fits the given subgroup of subjects, but not how well the classification generalizes to other subject populations. We counteract this by three approaches; (1) classification was performed on the second EEG recording, whereas the prediction was tested on the first EEG recording, (2) as the ultimate test we evaluated the prediction on subjects not included for classifier training (because not all subjects had a second EEG recording), and (3) we performed a half-split cross-validation. In the half-split cross-validation the sample was divided randomly in half several times (1000 iterations); the classifier was then trained on the first half, and the outcome was evaluated on the second half. We report the median outcome measures over these splits. Cross-validation gives an estimate of the classification performance on an “unknown” sample (Witten et al., 2011). However, cross-validation also suffers from lower *n* numbers, which means their outcome should be viewed as a conservative estimate of the average outcome.

### Statistics: group differences and correlations

We use non-parametric permutation tests based on median (Box and Andersen, 1955; Ernst, 2004) to test for differences between groups. Non-parametric tests are more robust toward non-normal data, but also often have lower power than parametric such as student's *t*-test. Confidence intervals (95%) were found using non-parametric bias corrected and accelerated bootstrap (*n* = 5000) (DiCiccio and Efron, 1996).

### Statistics: 2 × 2 table independence tests

To test for dependence of genotype, gender, and patient group we used Barnard's exact test, which is appropriate for low sample statistics compared with Chi-square test, and has better power compared with Fisher's exact test (Barnard, 1947).

### Statistics: multiple comparisons

Because we do large-scale mapping of biomarkers, we employ a lenient approach to multiple comparisons correction at the first level of analysis. This means that in the initial mapping of potential difference between the stable MCI and AD-converter groups, we only perform a binomial correction for the number of significant channels in each biomarker (Poil et al., 2011). We do not correct the *p-*values across different biomarkers. This approach is appropriate since this mapping of potential difference is only used to identify candidate biomarkers for the genetic search algorithm.

## Results

### Patient groups—age and gender

Initially 86 subjects (Age: 68.7 [66.5:71.3] years, median [95% confidence interval], age at first EEG, 58 males) were diagnosed with mild cognitive impairment (MCI). After 415 ± [393:478] days, 17 patients (9 males) had converted to Alzheimer's disease. After 709 ± [537:779] days (1.9 years) a total of 25 patients (14 males) had converted to AD (Age: 69 ± [67:72] years), 39 subjects (28 males) remained MCI (Age: 67 ± [65:71] years), 9 subjects (6 males) were diagnosed with subjective complaints (Age: 67 ± [46:73] years), and 13 patients (10 males) with other disorders (Age: 70 ± [61:74] years) (including frontal lobe and Vascular dementia). No significant difference was found in age and gender between stable MCI and AD-converters (Gender: Barnard's test, *p* = 0.16; Age: permutation test, *p* = 0.49) (Table 1). We only focus on the patients diagnosed with AD, and subjects remaining stable MCI. In the following we use the last diagnosis of the subjects for the definition of the MCI-stable and AD-converters groups.

### MMSE results

The MMSE score of the MCI-stable group (28 ± [27:29]) was not significantly different from the score from AD-converter group (27 ± [26:28]) at the intake test (permutation test, *p* = 0.8). At the follow up approximately 1 year later the stable MCI subjects remained at a stable MMSE score of 28 ± [26:29], whereas the MMSE score of the AD-converter group changed to 24 ± [22:24] (permutation test, *p* = 0.0044), which is also lower than the stable MCI group's MMSE scores (permutation test, *p* = 0.0002) (Table 1).

### APOE status

We observed a significantly higher frequency of E4 allele vs. no E4 allele in AD-converter vs. stable MCI (Barnard test, *p* < 0.01). Only 38% of MCI-stable compared to 64% of AD-converter group had more than one E4 allele (Table 2).

**Table 2:** **Overview of patient groups**.

**Patient group**	**Age [years]**	**MMSE 1st year**	**MMSE 2nd year**	**Number of APOE E4**
MCI-stable	67 ± [65:71]	28 ± [27:29]	28 ± [26:29]	15 out of 39
AD-convert	69 ± [67:72]	27 ± [26:28]	24 ± [22:24]	16 out of 25
Difference (*p*-value)	*p* = 0.49	*p* = 0.8	*p* = 0.0002	*p* < 0.01

### Single-biomarker logistic regression model of AD-converter vs. MCI-Stable

**Figure 3:**
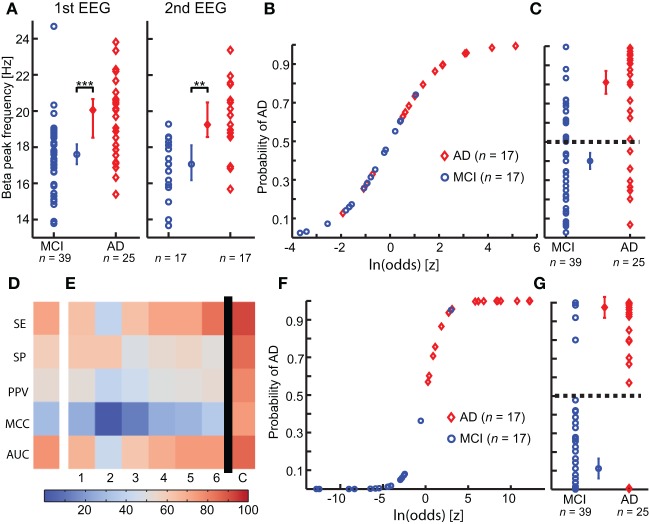
**Integration of multiple biomarkers using logistic regression improves the prediction of Alzheimer's disease at the MCI stage. (A)** A significant higher Beta peak frequency is observed in Alzheimer's disease converter group (AD) (red) compared with mild cognitive impairment stable group (MCI) (blue), in both first (left) and second (right) year EEG recording. (permutation test on median, binomial corrected, ^**^p < 0.005, ^***^p < 0.0005) (**B)** The logistic model is fitted to the second-year EEG recording. **(C)** The logistic model is used to predict outcome on the first year EEG recording. Separation plot of AD vs. MCI. **(D)** Outcome evaluation of beta peak frequency using five measures of classification power (warmer is better). SE, Sensitivity; SP, Specificity; PPV, Positive predictive value; MCC, Matthews Correlation Coefficient; AUC, area under the receiver operator curve. **(E)** Outcome evaluation as in **(C)**, but for the “optimal” biomarker set found using genetic search. The first six columns are for classification of the individual biomarkers separately. The last column is the combined classification outcome. We clearly see that the combined outcome is better than the classification using the individual biomarkers. 1, Peak width of dominant beta peak; 2, range of amplitude values in Beta (13–30 Hz); 3, Bandwidth of subject-specific Beta frequency; 4, Ratio between theta and alpha power; 5, alpha relative power (normalized with 1–45 Hz broadband); 6, Amplitude correlations with Cz in Beta (13–30 Hz); **(C)** Combined logistic classification using the biomarkers 1, 2, 3, 4, 5, and 6. **(F)** Logistic curve for combined classification based on first-year EEG. **(G)** Separation plot of MCI vs. AD in first EEG recording using combined classification based on second-year logistic regression coefficients. Note that the recordings used for training in F are different from those used for testing in **(G)**.

To show the principle of logistic regression modeling on a single biomarker, we chose the beta peak frequency, because this biomarker showed significantly lower values in MCI (MCI: 17.6 ± [16.8:18.2] Hz, *n* = 39) compared with the AD-converter group (AD: 19.6 ± [18.1:21.0] Hz, *n* = 25) (*p* < 0.0005) in the first measurement (Figure 3A), and also significantly lower values in MCI in the second measurement (MCI: 16.9 ± [16.0:17.8] Hz, *n* = 17; AD: 19.3 ± [18.6:20.6] Hz, *n* = 17, *p* < 0.005) (frequency values are averages across the significant channels) (Figure 3A).

We fitted a logistic regression model to the second EEG measurement (*n* = 17 in both groups, Figure 3B). The model classified the second measurements with a sensitivity (SE) of 76%, 76% specificity (SP), 76% positive predictive value (PPV), 0.5 Matthew correlation coefficient (MCC), and a positive likelihood ratio (PLR) of 3.3. Next, we used this logistic model to retrospectively classify the first EEG measurement (Figure 3C). The classification had a SE of 72%, 59% SP, 53% PPV, 0.3 MCC and a PLR of 1.8; thus, as expected, a worse classification power (MCI *n* = 39, AD *n* = 25) (Figure 3D).

### Multiple-biomarker logistic regression model of AD-converter vs. MCI-Stable

By combining several biomarkers it may be possible to obtain better classification power than the individual biomarkers alone (Schoonenboom et al., 2004; Buscema et al., 2007; Lehmann et al., 2007). However, it is not trivial which combinations of biomarkers are optimal, because of the high number of possible combinations. Here, we employ a genetic search approach and elastic net penalization to assists us in finding these optimal combinations (see Methods and Materials section).

The best set of biomarkers identified by the genetic search was (six biomarkers): Amplitude correlations with Cz in Beta (13–30 Hz), Bandwidth of subject-specific Beta frequency, Peak width of dominant beta peak, range of amplitude values in Beta (13–30 Hz), Ratio between theta and alpha power, and alpha relative power (normalized with 1–45 Hz broadband). The logistic regression training on this biomarker set using the second EEG data yielded a SE of 100%, 94% SP, 94% PPV, 0.94 MCC, and PLR of 17 (*n* = 17 in both groups).

**Figure 4:**
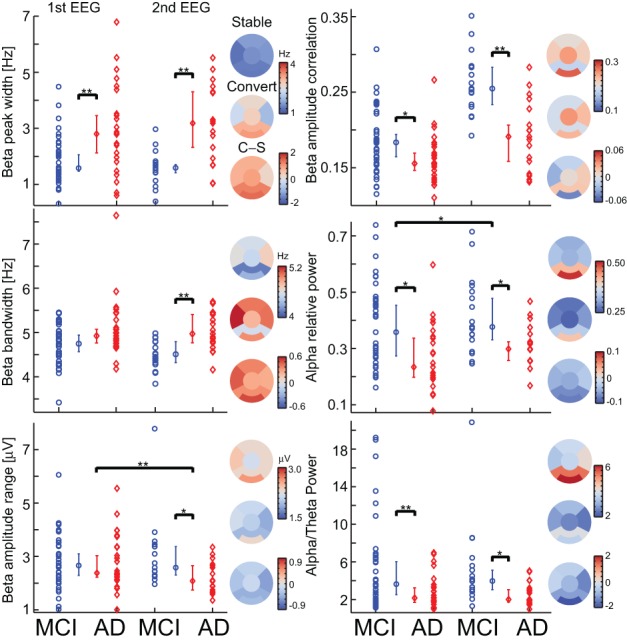
**Most biomarkers in the diagnostic index have differences between the MCI-stable (MCI) and AD-converter (AD) groups, and only two have longitudinal changes**. For each biomarker, a separation plot is shown for 1st EEG and 2nd EEG. The values are median across channels with significant differences between the MCI-stable (MCI) and AD-converter (AD) groups (Binomial corrected). Topographical plots are of 2nd EEG median value across subjects and channels in 6 regions; Frontal, left/right temporal, Central, Parietal, and Occipital. Asterisk indicates significant differences (permutation test on median, binomial corrected, ^*^p < 0.05, ^**^p < 0.005).

The retrospective testing on first-year data using the classifier model trained on the second-year data gave a SE of 92%, 85% SP, 79% PPV, 0.75 MCC, and PLR of 6 (MCI-stable, *n* = 39; AD-convert, *n* = 25) (Figures 3F,G; Table 3), which indicates that even at this very early stage differences between AD-converters and MCI-stable can be identified. However, since second-year and first-year data from the same subjects may be strongly correlated we also performed a classification test using only subjects that were not used for training the model (i.e., the subjects without a second EEG recording). We obtained a good classification with a SE of 88%, 82% SP, 64% PPV, 0.64 MCC and a PLR of 4.8 (MCI-stable, *n* = 22; AD-convert, *n* = 8), suggesting the diagnostic index can generally be used for these patient groups. Furthermore, we performed a half-split cross-validation (1000 iterations), with a SE of 75%, 63% SP, 52% PPV, 0.37 MCC, and a PLR of 2, an indication of the average outcome. As expected, the classification powers decrease; however, this is at least partly explained by the lower *n* number. However, the combined classification is still much better than prediction obtained on the individual biomarkers in the set (Figures 3E, 4). The best single biomarker in the biomarker set (based on sensitivity and specificity) was the peak width of the dominant Beta peak, with a SE of 64%, 62% SP, 52% PPV, 0.24 MCC, and a PLR of 1.7 (MCI-stable, *n* = 22; AD-convert, *n* = 8) (Table 3). The logistic regression fitting coefficients for the combined solution were; −2.9 for Amplitude correlations with Cz in Beta, 0.5 for bandwidth of subject specific Beta, 3.4 for Peak width of dominant beta peak, −0.6 for range of amplitude values in Beta, −2.3 for ratio between theta and alpha power, and −0.2 for alpha relative power. This means that the peak width of the dominant beta peak had the greatest influence on the outcome, followed by amplitude correlations with Cz.

**Table 3:** **Overview of classification results [classification based on testing subjects that were not used for training the classifier (MCI-stable, *n* = 22; AD-convert, *n* = 8)]**.

**Model**	**Sensitivity (%)**	**Specificity (%)**	**Positive predictive value (%)**	**Matthew correlation coefficient**	**Positive likelihood ratio**
Genetic search 6 biomarkers	88	82	64	0.64	4.8
Single best biomarker	64	62	52	0.24	1.7
Elastic-net 12 biomarkers	75	86	67	0.59	5.5

Taken together, our results show that it is possible to obtain a substantial synergistic effect from the integration of several biomarkers; however, they also show that it is not trivial to identify which combination of biomarkers is most optimal. The major issue with our genetic search is that from run to run we do not obtain the same solution, because the algorithm finds local maxima. We, therefore, employed an elastic net penalized logistic regression algorithm. This algorithm uses a penalization of the weights to optimize the set of biomarkers used for classification. The classification outcome from this algorithm is worse than genetic search optimized logistic regression, with a SE of 75%, 86% SP, 67% PPV, 0.59 MCC, and a PLR of 5.5 (MCI-stable, *n* = 22; AD-convert, *n* = 8) (Table 3) based on training on the second-year EEG and testing on the first-year recording of subjects (the test subjects were not used for training). The elastic net logistic regression combined 12 biomarkers (non-zero weights), namely; the amplitude correlations from Cz in Alpha (8–13 Hz) and Beta (13–30 Hz), the range of the generalized multifractal hurst exponent of the Delta (1–3 Hz) amplitude envelope, the Beta frequency, the power ratio between Gamma (30–45 Hz) and Delta (1–4 Hz), Alpha 1 (Individual Alpha frequency-4: Individual Alpha frequency-2) and Alpha (8–13), Alpha 1 and Beta (13–30 Hz), the spectral edge of the individualized beta-frequency range, the peak width of the beta peak, the second beta peak frequency, the stability of the Delta (1–3 Hz) frequency measured in windows of 5 s, and the Hjorth mobility parameter. The outcome evaluation still shows room for improvement, e.g., by including biomarkers from other modalities.

## Discussion

We addressed the challenge of predicting whether an MCI subject would convert to AD within 2 years. To this end, we explored the added value of integrating multiple EEG biomarkers into a diagnostic index using logistic regression in combination with either a genetic search or elastic-net penalization for biomarker selection. From an initial cohort of 86 subjects with mild cognitive impairment, 25 converted to Alzheimer's disease within 2 years. We showed how data mining of 177 EEG biomarkers could be used to identify a set of biomarkers that form a diagnostic index. The analysis was performed using the Neurophysiological Biomarker Toolbox (NBT, http://www.nbtwiki.net/) (Hardstone et al., 2012), which is specifically developed to support data mining and integration of large sets of biomarkers. We found that particularly biomarkers sensitive to changes in the beta frequency (13–30 Hz) band were optimal for classifying the very early EEG recordings of yet to be diagnosed AD patients.

### Classification based diagnostics

Previous studies have shown promise in using machine-learning algorithms to classify between MCI and AD based on EEG recordings (Huang et al., 2000; Bennys et al., 2001; Prichep et al., 2006; Buscema et al., 2007; Lehmann et al., 2007; Prichep, 2007; Rossini et al., 2008). A sensitivity of 89% and specificity of 95% were, e.g., found using the so-called IFAST model (Buscema et al., 2007; Rossini et al., 2008). However, these studies were based on training and testing on the same data, which makes it more difficult to judge the performance. Uniquely to the present study, we performed classification training on the second EEG recording, and retrospectively used this to perform prediction based on the first EEG recording from subjects not used for the training. We note, however, that the drawback of the present procedure is the low number of patients in the smallest patient group (i.e., the eight patients converting to AD) produced a fairly high error margin to the classification estimates (12.5%).

### Oscillations are involved in cognition

Empirical and theoretical evidence suggest that oscillations provide important systems-level mechanisms for normal brain function (Engel and Singer, 2001; Buzsáki and Draguhn, 2004; Axmacher et al., 2006; Klimesch et al., 2007; Palva and Palva, 2007, 2012; Lisman, 2010). For example, oscillations are involved in memory encoding (Raghavachari et al., 2001; Jensen et al., 2002), and are thought to provide a timing mechanism for spike-time dependent plasticity (Engel and Fries, 2010). It, therefore, seem plausible that if oscillations are abnormal in disorders such as MCI and AD, then cognition is also affected. Apart from relative Alpha power and the theta/alpha power ratio, which may reflect early changes toward the well-known slowing of the EEG in AD (Bennys et al., 2001; Rossini et al., 2006), our optimal set of biomarkers is derived from the Beta frequency band (13–30 Hz).

Beta-band changes have previously been observed in Alzheimer's disease, e.g., by a more anterior distribution (Huang et al., 2000). The larger width of the beta peak and bandwidth could potentially be linked with a less stable beta frequency, and, therefore, also a less efficient working memory (Kopell et al., 2011). Beta oscillations are believed to maintain the current sensorimotor and cognitive state (Engel and Fries, 2010). Activity in the beta-frequency range has also traditionally been linked with motor function. Interestingly, it has been found that motor performance is impaired in early-stage Alzheimer's disease but not in mild cognitive impairment (Sheridan et al., 2003; Pettersson et al., 2005), which is a potential explanation of the prominent role of beta-frequency changes in our data. Motor function, e.g., gait control, is a higher cognitive function requiring integration of several cognitive functions, as attention, planning (Hausdorff et al., 2005; Scherder et al., 2007), albeit unrelated to performance in memory tests (Hausdorff et al., 2005). Hyperexcitability of the motor cortex has also been observed in AD (Di Lazzaro et al., 2004), which our finding of higher beta frequency also suggests.

### EEG biomarkers as potential indicators of inflammation

The standard hypothesis of Alzheimer's disease is the amyloid cascade hypothesis stating that the cause of Alzheimer's should be found in the build up of amyloid and tangles (Hardy and Selkoe, 2002; Huang and Mucke, 2012). It has been hypothesized that Alzheimer's disease is initiated by a micro injury, presumable a vascular event, in the brain with subsequent activation of inflammatory responses that further leads to initiation of the amyloid deposition cycle (De la Torre, 2004; Herrup, 2010).

The theta/(lower alpha) power ratio has previously been associated with vascular damage in AD (Moretti et al., 2007b), and the delta (2–4 Hz) power has been associated with inflammation (Babiloni et al., 2009). EEG power and frequency in general has also been correlated with cerebral perfusion (O'Gorman et al., 2013), which is known to be reduced in Alzheimer's disease (De la Torre, 1999; Kogure et al., 2000; Murray et al., 2011). If we could detect early-stage changes using EEG, we would have a powerful tool that could detect Alzheimer's disease at a point where a possible therapy would be most efficient. Mouse models, e.g., show that Aβ-42 modifying therapy has limited effect after neurodegeneration has begun (Dubois et al., 2007; Sperling et al., 2011). Thus, meaning that diagnosing a patient based on neurodegeneration and cognitive decline may already be too late for a good treatment outcome because the brain damage has already occurred.

It has also been shown that the build-up of Aβ 42 influences synaptic transmission, and thus, potentially also give rise to further effects in the EEG (Palop and Mucke, 2009; Verret et al., 2012). Further hippocampal injections of amyloid β in rats have been shown to induce impaired memory performance combined with reduced hippocampal theta oscillations and less activity in GABAergic neurons (GABA, gamma-aminobutyric acid) (Villette et al., 2010). A recent suggestion for a potential improvement of Alzheimer's disease symptoms is transcranial direct current stimulation (tDCS) (Hansen, 2012). This method increased theta and alpha oscillations together with improved working memory performance (Zaehle et al., 2010). Interestingly, it has been suggested these effects may be caused by altered GABA concentration within the stimulated cortex, and potentially by an adjustment of the excitatory/inhibitory balance, which is disturbed in Alzheimer's disease (Di Lazzaro et al., 2004; Rossini et al., 2007; Stagg et al., 2009). This balance may be directly linked to EEG biomarkers that have been shown sensitive to Alzheimer's disease (Montez et al., 2009; Poil et al., 2011, 2012). It thus seems that EEG biomarkers may be sensitive to underlying pathophysiology of AD.

### Outlook

We here showed that exploratory data mining and integration of multiple biomarkers might yield many exciting results on the large databases of neuroscience data build up over the years. These studies may identify hidden structures (see schematic Figure 1C) and be beneficial for both pre-clinical and clinical research. With recent developments in automatic cleaning of EEG this analysis may potentially be performed immediately after the recording (Nolan et al., 2010; Mognon et al., 2011). This together with the non-invasive character of EEG could make a diagnostic index using EEG biomarkers a powerful tool to support the early-stage clinical assessment. EEG biomarkers, apart from being non-invasive and relative inexpensive, have the advantage of monitoring brain activity in real time, and thus potentially able to identify tiny changes in ongoing cognition. However, we believe the best diagnostic/prognostic performance is achieved if EEG biomarkers are combined with information from other modalities. Future studies should specifically study how the synergistic information of integrative biomarkers can be improved further by the incorporation of different classes of biomarkers, which could range from cognitive markers (Tabert et al., 2006), functional connectivity markers (Stam et al., 2006, 2007), coherence, synchronization, and topographical location markers (Huang et al., 2000; Stam et al., 2005; Rossini et al., 2006) to questionnaire data providing quantitative data on the mental state of the patients during the resting-state EEG recording (Diaz et al., 2013). Improvement in algorithms used for pre-selecting biomarkers could, e.g., be based on measures of interrelatedness between biomarkers or taking scalp topographies into account as opposed to the averaged channel biomarker values used here. We believe the Neurophysiological Biomarker toolbox provides a promising framework for these studies. This could give rise to a better integrative understanding of biomarkers involved with Alzheimer's disease and brain disorders in general (Searls, 2005; Dubois et al., 2007; Schneider, 2010).

### Conflict of interest statement

The authors declare that the research was conducted in the absence of any commercial or financial relationships that could be construed as a potential conflict of interest.

## References

[B1] Albert M. S. DeKosky S. T. Dickson D. Dubois B. Feldman H. H. Fox N. C. (2011). The diagnosis of mild cognitive impairment due to Alzheimer's disease: recommendations from the National Institute on Aging and Alzheimer's Association workgroup. Alzheimers Dement. 7, 270–279 10.1016/j.jalz.2011.03.008 21514249 PMC3312027

[B2] Antila K. Lötjönen J. Thurfjell L. Laine J. Massimini M. Rueckert D. (2013). The PredictAD project: development of novel biomarkers and analysis software for early diagnosis of the Alzheimer's disease. Interface Focus 3 10.1098/rsfs.2012.0072 PMC3638476 24427524

[B3] Axmacher N. Mormann F. Fernández G. Elger C. E. Fell J. (2006). Memory formation by neuronal synchronization. Brain Res. Rev. 52, 170–182 10.1016/j.brainresrev.2006.01.007 16545463

[B4] Babiloni C. Binetti G. Cassetta E. Forno G. D. Percio C. D. Ferreri F. (2006). Sources of cortical rhythms change as a function of cognitive impairment in pathological aging: a multicenter study. Clin. Neurophysiol. 117, 252–268 10.1016/j.clinph.2005.09.019 16377238

[B5] Babiloni C. Frisoni G. B. Del Percio C. Zanetti O. Bonomini C. Cassetta E. (2009). Ibuprofen treatment modifies cortical sources of EEG rhythms in mild Alzheimer's disease. Clin. Neurophysiol. 120, 709–718 10.1016/j.clinph.2009.02.005 19324592

[B6] Babiloni C. Frisoni G. B. Vecchio F. Lizio R. Pievani M. Cristina G. (2011). Stability of clinical condition in mild cognitive impairment is related to cortical sources of alpha rhythms: An electroencephalographic study. Hum. Brain Mapp. 32, 1916–1931 10.1002/hbm.21157 21181798 PMC6869969

[B6a] Baldi P. Brunak S. Chauvin Y. Andersen C. A. F. Nielsen H. (2000). Assessing the accuracy of prediction algorithms for classification: an overview. Bioinformatics rev. 16, 412–424 10.1093/bioinformatics/16.5.412 10871264

[B7] Barnard G. A. (1947). Significance tests for 2X2 tables. Biometrika 34, 123–138 20287826 10.1093/biomet/34.1-2.123

[B8] Bennys K. Rondouin G. Vergnes C. Touchon J. (2001). Diagnostic value of quantitative EEG in Alzheimer's disease. Neurophysiol. Clin. Neurophysiol. 31, 153–160 10.1016/S0987-7053(01)00254-4 11488226

[B9] Blennow K. de Leon M. J. Zetterberg H. (2006). Alzheimer's disease. Lancet 368, 387–403 10.1016/S0140-6736(06)69113-7 16876668

[B10] Box G. E. Andersen S. L. (1955). Permutation theory in the derivation of robust criteria and the study of departures from assumption. J. R. Stat. Soc. Ser. B Methodol. 17, 1–34

[B11] Buscema M. Rossini P. Babiloni C. Grossi E. (2007). The IFAST model, a novel parallel nonlinear EEG analysis technique, distinguishes mild cognitive impairment and Alzheimer's disease patients with high degree of accuracy. Artif. Intell. Med. 40, 127–141 10.1016/j.artmed.2007.02.006 17466496

[B12] Buzsáki G. Draguhn A. (2004). Neuronal oscillations in cortical networks. Science 304, 1926–1929 10.1126/science.1099745 15218136

[B13] Cardoso J. F. Souloumiac A. (1993). Blind beamforming for non-Gaussian signals. IEEE Proc. F 140, 362–370 10.1049/ip-f-2.1993.005419507966

[B14] De la Torre J. C. (1999). Critical threshold cerebral hypoperfusion causes Alzheimer's disease. Acta Neuropathol. 98, 1–8 10.1007/s004010051044 10412794

[B15] De la Torre J. C. (2004). Is Alzheimer's disease a neurodegenerative or a vascular disorder. Data, dogma, and dialectics. Lancet Neurol. 3, 184–190 10.1016/S1474-4422(04)00683-0 14980533

[B16] Di Lazzaro V. Oliviero A. Pilato F. Saturno E. Dileone M. Marra C. (2004). Motor cortex hyperexcitability to transcranial magnetic stimulation in Alzheimer's disease. J. Neurol. Neurosurg. Psychiatr. 75, 555–559 10.1136/jnnp.2003.018127 15026495 PMC1739006

[B17] Diaz B. A. van der Sluis S. Moens S. Benjamins J. S. Migliorati F. Stoffers D. (2013). The Amsterdam Resting-State Questionnaire reveals multiple phenotypes of resting-state cognition. Front. Hum. Neurosci. 7:446 10.3389/fnhum.2013.00446 23964225 PMC3737475

[B18] DiCiccio T. J. Efron B. (1996). Bootstrap confidence intervals. Stat. Sci. 11, 189–212

[B19] Dubois B. Feldman H. H. Jacova C. DeKosky S. T. Barberger-Gateau P. Cummings J. (2007). Research criteria for the diagnosis of Alzheimer's disease: revising the NINCDS–ADRDA criteria. Lancet Neurol. 6, 734–746 10.1016/S1474-4422(07)70178-3 17616482

[B20] Engel A. K. Fries P. (2010). Beta-band oscillations—signalling the status quo. Curr. Opin. Neurobiol. 20, 156–165 10.1016/j.conb.2010.02.015 20359884

[B21] Engel A. K. Singer W. (2001). Temporal binding and the neural correlates of sensory awareness. Trends Cogn. Sci. 5, 16–25 11164732 10.1016/s1364-6613(00)01568-0

[B22] Ernst M. D. (2004). Permutation methods: a basis for exact inference. Stat. Sci. 19, 676–685 10.1214/088342304000000396

[B23] Flicker C. Ferris S. H. Reisberg B. (1991). Mild cognitive impairment in the elderly. Neurology 41, 1006–1006 10.1212/WNL.41.7.1006 2067629

[B24] Folstein M. F. Folstein S. E. McHugh P. R. (1975). “Mini-mental state”. A practical method for grading the cognitive state of patients for the clinician. J. Psychiatr. Res. 12, 189–198 10.1016/0022-3956(75)90026-6 1202204

[B25] Friedman J. Hastie T. Tibshirani R. (2010). Regularization paths for generalized linear models via coordinate descent. J. Stat. Softw. 33, 1–22 20808728 PMC2929880

[B26] Gauthier S. Reisberg B. Zaudig M. Petersen R. C. Ritchie K. Broich K. (2006). Mild cognitive impairment. Lancet 367, 1262–1270 10.1016/S0140-6736(06)68542-5 16631882

[B27] Goncharova I. I. Barlow J. S. (1990). Changes in EEG mean frequency and spectral purity during spontaneous alpha blocking. Electroencephalogr. Clin. Neurophysiol. 76, 197–204 10.1016/0013-4694(90)90015-C 1697252

[B28] Hansen N. (2012). Action mechanisms of transcranial direct current stimulation in Alzheimer's disease and memory loss. Front. Psychiatry 3:48 10.3389/fpsyt.2012.00048 22615703 PMC3351674

[B29] Hardstone R. Poil S.-S. Schiavone G. Jansen R. Nikulin V. V. Mansvelder H. D. (2012). Detrended fluctuation analysis: a scale-free view on neuronal oscillations. Front. Physiol. 3:450 10.3389/fphys.2012.00450 23226132 PMC3510427

[B30] Hardy J. Selkoe D. J. (2002). The amyloid hypothesis of Alzheimer's disease: progress and problems on the road to therapeutics. Science 297, 353–356 10.1126/science.1072994 12130773

[B31] Hausdorff J. M. Yogev G. Springer S. Simon E. S. Giladi N. (2005). Walking is more like catching than tapping: gait in the elderly as a complex cognitive task. Exp. Brain Res. 164, 541–548 10.1007/s00221-005-2280-3 15864565

[B32] Herrup K. (2010). Reimagining Alzheimer's disease—an age-based hypothesis. J. Neurosci. 30, 16755–16762 10.1523/JNEUROSCI.4521-10.2010 21159946 PMC3004746

[B33] Hjorth B. (1970). EEG analysis based on time domain properties. Electroencephalogr. Clin. Neurophysiol. 29, 306–310 10.1016/0013-4694(70)90143-4 4195653

[B34] Huang C. Wahlund L. O. Dierks T. Julin P. Winblad B. Jelic V. (2000). Discrimination of Alzheimer's disease and mild cognitive impairment by equivalent EEG sources: a cross-sectional and longitudinal study. Clin. Neurophysiol. 111, 1961–1967 10.1016/S1388-2457(00)00454-5 11068230

[B35] Huang L. Mucke L. (2012). Alzheimer mechanisms and therapeutic strategies. Cell 148, 1204–1222 10.1016/j.cell.2012.02.040 22424230 PMC3319071

[B36] Hughes C. P. Berg L. Danziger W. L. Coben L. A. Martin R. L. (1982). A new clinical scale for the staging of dementia. Br. J. Psychiatry 140, 566–572 10.1192/bjp.140.6.566 7104545

[B37] Ihlen E. A. F. (2012). Introduction to multifractal detrended fluctuation analysis in matlab. Front. Physiol. 3:141 10.3389/fphys.2012.00141 22675302 PMC3366552

[B38] Jelic V. Johansson S. E. Almkvist O. Shigeta M. Julin P. Nordberg A. (2000). Quantitative electroencephalography in mild cognitive impairment: longitudinal changes and possible prediction of Alzheimer's disease. Neurobiol. Aging 21, 533–540 10.1016/S0197-4580(00)00153-6 10924766

[B39] Jelic V. Shigeta M. Julin P. Almkvist O. Winblad B. Wahlund L. O. (1996). Quantitative electroencephalography power and coherence in Alzheimer's disease and mild cognitive impairment. Dement. Geriatr. Cogn. Disord. 7, 314–323 10.1159/000106897 8915037

[B40] Jensen O. Gelfand J. Kounios J. Lisman J. E. (2002). Oscillations in the alpha band (9–12 Hz) increase with memory load during retention in a short-term memory task. Cereb. Cortex 12, 877–882 10.1093/cercor/12.8.877 12122036

[B41] Kantelhardt J. W. Zschiegner S. A. Koscielny-Bunde E. Havlin S. Bunde A. Stanley H. E. (2002). Multifractal detrended fluctuation analysis of nonstationary time series. Phys. Stat. Mech. Appl. 316, 87–114 10.1016/S0378-4371(02)01383-3

[B42] Klimesch W. (1999). EEG alpha and theta oscillations reflect cognitive and memory performance: a review and analysis. Brain Res. Rev. 29, 169–195 10.1016/S0165-0173(98)00056-3 10209231

[B43] Klimesch W. Sauseng P. Hanslmayr S. (2007). EEG alpha oscillations: the inhibition–timing hypothesis. Brain Res. Rev. 53, 63–88 10.1016/j.brainresrev.2006.06.003 16887192

[B44] Kogure D. Matsuda H. Ohnishi T. Asada T. Uno M. Kunihiro T. (2000). Longitudinal evaluation of early Alzheimer's disease using brain perfusion SPECT. J. Nucl. Med. 41, 1155 10914904

[B45] Kopell N. Whittington M. A. Kramer M. A. (2011). Neuronal assembly dynamics in the beta1 frequency range permits short-term memory. Proc. Natl. Acad. Sci. U.S.A. 108, 3779–3784 10.1073/pnas.1019676108 21321198 PMC3048142

[B46] Koza J. Poli R. (2005). Genetic programming, in Search Methodologies, (Heidelberg: Springer), 127–164

[B47] Kwak Y. T. (2006). Quantitative EEG findings in different stages of Alzheimer's disease. J. Clin. Neurophysiol. 23, 457 10.1097/01.wnp.0000223453.47663.63 17016157

[B48] Lehmann C. Koenig T. Jelic V. Prichep L. John R. E. Wahlund L. O. (2007). Application and comparison of classification algorithms for recognition of Alzheimer's disease in electrical brain activity (EEG). J. Neurosci. Methods 161, 342–350 10.1016/j.jneumeth.2006.10.023 17156848

[B49] Linkenkaer-Hansen K. Nikouline V. V. Palva J. M. Ilmoniemi R. J. (2001). Long-range temporal correlations and scaling behavior in human brain oscillations. J. Neurosci. 21, 1370 11160408 10.1523/JNEUROSCI.21-04-01370.2001 PMC6762238

[B50] Lisman J. (2010). Working memory: the importance of theta and gamma oscillations. Curr. Biol. 20, R490–R492 10.1016/j.cub.2010.04.011 20541499

[B51] Luckhaus C. Grass-Kapanke B. Blaeser I. Ihl R. Supprian T. Winterer G. (2008). Quantitative EEG in progressing vs stable mild cognitive impairment (MCI): results of a 1-year follow-up study. Int. J. Geriatr. Psychiatry 23, 1148–1155 10.1002/gps.2042 18537220

[B52] McKhann G. Drachman D. Folstein M. Katzman R. Price D. Stadlan E. M. (1984). Clinical diagnosis of Alzheimer's disease. Neurology 34, 939–939 10.1212/WNL.34.7.939 6610841

[B53] Mognon A. Jovicich J. Bruzzone L. Buiatti M. (2011). ADJUST: an automatic EEG artifact detector based on the joint use of spatial and temporal features. Psychophysiology 48, 229–240 10.1111/j.1469-8986.2010.01061.x 20636297

[B54] Montez T. Poil S.-S. Jones B. F. Manshanden I. Verbunt J. Van Dijk B. W. (2009). Altered temporal correlations in parietal alpha and prefrontal theta oscillations in early-stage Alzheimer disease. Proc. Natl. Acad. Sci. U.S.A. 106, 1614 10.1073/pnas.0811699106 19164579 PMC2635782

[B55] Moretti D. V. Frisoni G. B. Fracassi C. Pievani M. Geroldi C. Binetti G. (2011). MCI patients' EEGs show group differences between those who progress and those who do not progress to AD. Neurobiol. Aging 32, 563–571 10.1016/j.neurobiolaging.2009.04.003 20022139

[B56] Moretti D. V. Miniussi C. Frisoni G. B. Geroldi C. Zanetti O. Binetti G. (2007a) Hippocampal atrophy and EEG markers in subjects with mild cognitive impairment. Clin. Neurophysiol. 118, 2716–2729 10.1016/j.clinph.2007.09.059 17977786

[B57] Moretti D. V. Miniussi C. Frisoni G. Zanetti O. Binetti G. Geroldi C. (2007b) Vascular damage and EEG markers in subjects with mild cognitive impairment. Clin. Neurophysiol. 118, 1866–1876 10.1016/j.clinph.2007.05.009 17576096

[B58] Moretti D. V. Pievani M. Fracassi C. Geroldi C. Calabria M. De Carli C. S. (2008). Brain vascular damage of cholinergic pathways and EEG markers in mild cognitive impairment. J. Alzheimers Dis. 15, 357–372 18997289 10.3233/jad-2008-15302

[B59] Murray I. V. J. Proza J. F. Sohrabji F. Lawler J. M. (2011). Vascular and metabolic dysfunction in Alzheimer's disease: a review. Exp. Biol. Med. 236, 772–782 10.1258/ebm.2011.010355 21680755

[B60] Nolan H. Whelan R. Reilly R. B. (2010). FASTER: fully automated statistical thresholding for EEG artifact rejection. J. Neurosci. Methods 192, 152–162 10.1016/j.jneumeth.2010.07.015 20654646

[B61] O'Gorman R. L. Poil S.-S. Brandeis D. Klaver P. Bollmann S. Ghisleni C. (2013). Coupling between resting cerebral perfusion and EEG. Brain Topogr. 26, 442–457 10.1007/s10548-012-0265-7 23160910

[B62] Palop J. J. Mucke L. (2009). Epilepsy and cognitive impairments in alzheimer disease. Arch. Neurol. 66, 435–440 10.1001/archneurol.2009.15 19204149 PMC2812914

[B63] Palva S. Palva J. M. (2007). New vistas for Alpha-frequency band oscillations. Trends Neurosci. 30, 150–158 10.1016/j.tins.2007.02.001 17307258

[B64] Palva S. Palva J. M. (2012). Discovering oscillatory interaction networks with M/EEG: challenges and breakthroughs. Trends Cogn. Sci. Available online at*:* http://www.sciencedirect.com/science/article/pii/S1364661312000472 [Accessed November 3, 2012]. 10.1016/j.tics.2012.02.004 22440830

[B65] Petersen R. C. Smith G. E. Waring S. C. Ivnik R. J. Tangalos E. G. Kokmen E. (1999). Mild cognitive impairment: clinical characterization and outcome. Arch. Neurol. 56, 303 10.1001/archneur.56.3.303 10190820

[B66] Pettersson A. F. Olsson E. Wahlund L. O. (2005). Motor function in subjects with mild cognitive impairment and early Alzheimer's disease. Dement. Geriatr. Cogn. Disord. 19, 299–304 10.1159/000084555 15785030

[B67] Poil S.-S. Hardstone R. Mansvelder H. D. Linkenkaer-Hansen K. (2012). Critical-state dynamics of avalanches and oscillations jointly emerge from balanced excitation/inhibition in neuronal networks. J. Neurosci. 32, 9817–9823 10.1523/JNEUROSCI.5990-11.2012 22815496 PMC3553543

[B68] Poil S.-S. Jansen R. van Aerde K. Timmerman J. Brussaard A. B. Mansvelder H. D. (2011). Fast network oscillations *in vitro* exhibit a slow decay of temporal auto-correlations. Eur. J. Neurosci. 34, 394–403 10.1111/j.1460-9568.2011.07748.x 21692883

[B69] Poil S.-S. van Ooyen A. Linkenkaer-Hansen K. (2008). Avalanche dynamics of human brain oscillations: relation to critical branching processes and temporal correlations. Hum. Brain Mapp. 29, 770–777 10.1002/hbm.20590 18454457 PMC6871218

[B70] Prichep L. S. (2007). Quantitative EEG and electromagnetic brain imaging in aging and in the evolution of dementia. Ann. N.Y. Acad. Sci. 1097, 156–167 10.1196/annals.1379.008 17413018

[B71] Prichep L. S. John E. R. Ferris S. H. Rausch L. Fang Z. Cancro R. (2006). Prediction of longitudinal cognitive decline in normal elderly with subjective complaints using electrophysiological imaging. Neurobiol. Aging 27, 471–481 10.1016/j.neurobiolaging.2005.07.021 16213630

[B72] Prins N. D. Visser P. J. Scheltens P. (2010). Can novel therapeutics halt the amyloid cascade. Alzheimers Res. Ther. 2. Available online at: www.biomedcentral.com/content/pdf/alzrt28.pdf (Accessed June 19, 2012). 10.1186/alzrt28 PMC2876783 20388189

[B73] Raghavachari S. Kahana M. J. Rizzuto D. S. Caplan J. B. Kirschen M. P. Bourgeois B. (2001). Gating of human theta oscillations by a working memory task. J. Neurosci. 21, 3175–3183 11312302 10.1523/JNEUROSCI.21-09-03175.2001 PMC6762557

[B74] Rombouts S. A. R. Barkhof F. Goekoop R. Stam C. J. Scheltens P. (2005). Altered resting state networks in mild cognitive impairment and mild Alzheimer's disease: an fMRI study. Hum. Brain Mapp. 26, 231–239 10.1002/hbm.20160 15954139 PMC6871685

[B75] Rossini P. Buscema M. Capriotti M. Grossi E. Rodriguez G. Del Percio C. (2008). Is it possible to automatically distinguish resting EEG data of normal elderly vs. mild cognitive impairment subjects with HIGH degree of accuracy? Clin. Neurophysiol. 119, 1534–1545 10.1016/j.clinph.2008.03.026 18485814

[B76a] Rossini P. M. Rossi S. Babiloni C. Polich J. (2007). Clinical neurophysiology of aging brain: From normal aging to neurodegeneration. Prog. Neurobiol. 83, 375–400 10.1016/j.pneurobio.2007.07.010 17870229

[B76] Rossini P. Del Percio C. Pasqualetti P. Cassetta E. Binetti G. Dal Forno G. (2006). Conversion from mild cognitive impairment to Alzheimer's disease is predicted by sources and coherence of brain electroencephalography rhythms. Neuroscience 143, 793–803 10.1016/j.neuroscience.2006.08.049 17049178

[B77] Scherder E. Eggermont L. Swaab D. Van Heuvelen M. Kamsma Y. De Greef M. (2007). Gait in ageing and associated dementias; its relationship with cognition. Neurosci. Biobehav. Rev. 31, 485–497 10.1016/j.neubiorev.2006.11.007 17306372

[B78] Schneider L. S. (2010). Organising the language of Alzheimer's disease in light of biomarkers. Lancet Neurol. 9, 1044–1045 10.1016/S1474-4422(10)70246-5 20934913

[B79] Schoonenboom N. S. M. Pijnenburg Y. A. L. Mulder C. Rosso S. M. Van Elk E. J. Van Kamp G. J. (2004). Amyloid Beta (1–42) and phosphorylated tau in CSF as markers for early-onset Alzheimer disease. Neurology 62, 1580–1584 10.1212/01.WNL.0000123249.58898.E0 15136685

[B80] Searls D. B. (2005). Data integration: challenges for drug discovery. Nat. Rev. Drug Discov. 4, 45–58 10.1038/nrd1608 15688072

[B81] Sheridan P. L. Solomont J. Kowall N. Hausdorff J. M. (2003). Influence of executive function on locomotor function: divided attention increases gait variability in Alzheimer's disease. J. Am. Geriatr. Soc. 51, 1633–1637 10.1046/j.1532-5415.2003.51516.x 14687395

[B82] Sperling R. A. Aisen P. S. Beckett L. A. Bennett D. A. Craft S. Fagan A. M. (2011). Toward defining the preclinical stages of Alzheimer's disease: recommendations from the National Institute on Aging and the Alzheimer's Association workgroup. Alzheimers Dement. 7, 280–292 10.1016/j.jalz.2011.03.003 21514248 PMC3220946

[B83] Stagg C. J. Best J. G. Stephenson M. C. O'Shea J. Wylezinska M. Kincses Z. T. (2009). Polarity-sensitive modulation of cortical neurotransmitters by transcranial stimulation. J. Neurosci. 29, 5202–5206 19386916 10.1523/JNEUROSCI.4432-08.2009 PMC6665468

[B84] Stam C. J. Jones B. F. Manshanden I. Van Cappellen van Walsum A. M. Montez T. Verbunt J. P. A. (2006). Magnetoencephalographic evaluation of resting-state functional connectivity in Alzheimer's disease. Neuroimage 32, 1335–1344 10.1016/j.neuroimage.2006.05.033 16815039

[B85] Stam C. J. Jones B. F. Nolte G. Breakspear M. Scheltens P. (2007). Small-world networks and functional connectivity in Alzheimer's disease. Cereb. Cortex 17, 92–99 10.1093/cercor/bhj127 16452642

[B86] Stam C. J. Montez T. Jones B. F. Rombouts S. Van Der Made Y. Pijnenburg Y. A. L. (2005). Disturbed fluctuations of resting state EEG synchronization in Alzheimer's disease. Clin. Neurophysiol. 116, 708–715 10.1016/j.clinph.2004.09.022 15721085

[B87] Stam C. J. Van der Made Y. Pijnenburg Y. A. L. Scheltens P. H. (2003). EEG synchronization in mild cognitive impairment and Alzheimer's disease. Acta Neurol. Scand. 108, 90–96 10.1034/j.1600-0404.2003.02067.x 12859284

[B88] Tabert M. H. Manly J. J. Liu X. Pelton G. H. Rosenblum S. Jacobs M. (2006). Neuropsychological prediction of conversion to Alzheimer disease in patients with mild cognitive impairment. Arch. Gen. Psychiatry 63, 916 10.1001/archpsyc.63.8.916 16894068

[B89] Van Aerde K. I. Mann E. O. Canto C. B. Heistek T. S. Linkenkaer-Hansen K. Mulder A. B. (2009). Flexible spike timing of layer 5 neurons during dynamic beta oscillation shifts in rat prefrontal cortex. J. Physiol. 587, 5177–5196 10.1113/jphysiol.2009.178384 19752121 PMC2790257

[B90] Verret L. Mann E. O. Hang G. B. Barth A. M. I. Cobos I. Ho K. (2012). Inhibitory interneuron deficit links altered network activity and cognitive dysfunction in alzheimer model. Cell 149, 708–721 10.1016/j.cell.2012.02.046 22541439 PMC3375906

[B91] Villette V. Poindessous-Jazat F. Simon A. Clement L. Roullot E. Bellessort B. (2010). Decreased rhythmic GABAergic septal activity and memory-associated theta oscillations after hippocampal amyloid-beta pathology in the rat. J. Neurosci. 30, 10991–11003 10.1523/JNEUROSCI.6284-09.2010 20720106 PMC6633464

[B92] Vural C. Yildiz M. (2010). Determination of sleep stage separation ability of features extracted from EEG signals using principle component analysis. J. Med. Syst. 34, 83–89 10.1007/s10916-008-9218-9 20192058

[B93] Wackermann J. (1999). Towards a quantitative characterisation of functional states of the brain: from the non-linear methodology to the global linear description. Int. J. Psychophysiol. 34, 65–80 10.1016/S0167-8760(99)00038-0 10555875

[B94] Witten I. H. Frank E. Hall M. A. (2011). Data Mining: Practical Machine Learning Tools and Techniques. San Francisco: Morgan Kaufmann

[B95] Zaehle T. Rach S. Herrmann C. S. (2010). Transcranial alternating current stimulation enhances individual alpha activity in human EEG. PLoS ONE 5:e13766 10.1371/journal.pone.0013766 21072168 PMC2967471

[B96] Zou H. Hastie T. (2005). Regularization and variable selection via the elastic net. J. R. Stat. Soc. Ser. B Stat. Methodol. 67, 301–320 10.1111/j.1467-9868.2005.00503.x

[B97] Zviling M. Leonov H. Arkin I. T. (2005). Genetic algorithm-based optimization of hydrophobicity tables. Bioinformatics 21, 2651–2656 10.1093/bioinformatics/bti405 15797910

